# Non-Pharmacological Interventions for the Management of Chronic Health Conditions and Non-Communicable Diseases

**DOI:** 10.3390/ijerph19148536

**Published:** 2022-07-13

**Authors:** Carmina Castellano-Tejedor

**Affiliations:** 1 Psynaptic, Psicología y Servicios Científicos y Tecnológicos S.L.P., 08192 Barcelona, Spain; info@psynaptic.es; 2 GIES Research Group, Basic Psychology Department, Autonomous University of Barcelona, 08192 Barcelona, Spain; 3 Research Group on Aging, Frailty and Care Transitions in Barcelona (REFiT BCN), Parc Sanitari Pere Virgili & Vall d’Hebron Research Institute (VHIR), 08023 Barcelona, Spain

## 1. The Conceptualisation of Non-Pharmacological Interventions

A chronic health condition has been defined by the World Health Organization (WHO) as a disease being of long duration, generally slow in progression and not passed from person to person; that is to say, a non-communicable disease (NCD) [1]. The Global Burden of Disease Study 2013 analysed 35,620 distinct data sources to document and calculate estimates for 301 diseases and injuries and 2337 sequelae [2]. Results revealed a substantial increase in the years lived with a disability (YLD) from 1990 to 2013, reaching up to 42.3% [2]. Findings were overwhelming due to NCDs, with no infectious diseases in the top 20 leading causes of YLDs globally in 2013. A few years before, in 2011, The Organisation for Economic Co-operation and Development (OECD) countries spent an average of 9.3% of their gross domestic product (GDP) on health care, with the United States being the highest spender, devoting 17.7% of GDP to health care [3]. The eight other countries profiled in this report have lower expenditure levels, ranging from 7.4% GDP in Korea to 11.9% GDP in the Netherlands. These costs include public and private spending on health care and capital investment in health care infrastructure. In addition, it is important to emphasise that if care provision falls upon families, personal and economic costs also multiply and become significant. It is important to understand how informal caregivers—usually family members—experience chronic conditions and NCDs, considering the possibilities of providing daily care according to the different and changeable needs that might emerge and how they could manage to maintain care over time. In this sense, approximately 50 million caregivers in North America provide care to a family member or a friend for issues related to chronic illness, disability, or ageing [4]. The annual economic value of caregivers’ unpaid care (on average 10–30 h per week) is estimated to be $450 billion in the USA and at least $26 billion in Canada [4]. With higher life expectancy and the increasing prevalence of chronic illness, the need for caregivers continues to grow and supporting them in maintaining their critical roles is imperative.

With this scenario, in recent years, Non-Pharmacological Interventions (NPIs) have attracted a lot of attention in the health care community. As defined by the Plateforme CEPS in 2017 [5]: “NPIs are science-based and non-invasive interventions for human health. They aim to prevent, treat, or cure health problems. NPIs may consist of products, methods, programs or services whose contents are known by users. They are linked to biological and/or psychological processes identified in clinical studies. NPIs have a measurable impact on health, quality of life, behavioural and socioeconomic markers. Their implementation requires relational, communicational and ethical skills”. Therefore, NPIs cannot rely on a unique professional discipline or field of knowledge to describe them (i.e., psychotherapy, occupational therapy, dietary supplement, adapted physical activity, e-health solutions, etc.). NPIs require access to a more concrete level of description where each NPI can be evaluated by science, monitored by professionals and explained accordingly to the patient to get their collaboration and active participation. The Plateforme CEPS [5] was founded in 2011 and is an Academic Collaborative Platform of methodology experts in clinical non-pharmacological research. Its mission is to foster the monitoring, design, implementation and publication of interventional studies dedicated to assessing NPIs’ efficacy. Despite great advances and the increasing corpus of scientific literature, more research from a multidisciplinary approach —biology, human & health sciences, technological engineering, mathematics, economics, political science, history or even philosophy—is required to further delve into this field of knowledge and to improve the quality of NPI studies for different health conditions. NPIs are commonly associated with non-acute settings, despite not being reduced to them. One broad classification may be as follows:

1. Psychological interventions: including psychoeducation/health education, stress management and relaxation techniques (i.e., Jacobson’s relaxation technique, meditation, mindfulness-based stress reduction, etc.), hypnosis, Eye Movement Desensitisation and Reprocessing, and cognitive–behavioural interventions (i.e., problem solving, planning, cognitive restructuring, etc.).
2. Acupuncture, Electroacupuncture, and Acupressure.
3. Biofeedback, transcutaneous electrical nerve stimulation (TENS) and other neuro and physical stimulation techniques carried out with devices.
4. Physical therapies (mainly including physiotherapy, massage, heat/cold, osteopathy, and chiropractic, among others).

An NPIs’ taxonomy proposed by the Plateforme CEPS [5], includes five categories:

5. Psychological Health Interventions: from prevention programs to psychotherapy interventions (i.e., Art Therapy, Health Education, Psychotherapy, Zootherapy).
6. Physical Health Interventions: from manual therapy to therapeutic physical activity programs (i.e., Physical Activity, Hortitherapy, Physiotherapy, Manual Therapy, Thermalism).
7. Nutritional Health Interventions: from supplementary food products to diet interventions (Dietary Supplements, Nutritional Therapy).
8. Digital Health Interventions: from health wearable and handheld devices to health coaching programs (i.e., eHealth Devices, Therapeutic Games, Virtual Reality Therapy).
9. Other Health Interventions: from phytotherapy to aromatherapy (i.e., Ergonomic tools, Phytotherapy, Cosmetic Therapy, Wave Therapy, Lithotherapy).

Although the Plateforme CEPS defined NPIs since 2017, other equivalent definitions were mentioned earlier, such as Complementary and Alternative Medicine (CAM), Integrative Medicine and/or Traditional Medicine [5]. The WHO defines traditional medicine as the sum of the knowledge, skill, and practices based on the theories, beliefs, and experiences indigenous to different cultures, whether explicable or not, used in the maintenance of health as well as in the prevention, diagnosis, improvement or treatment of physical and mental illness [6]. The WHO also proposes the definition of CAM as a broad set of healthcare practices that are not part of that country’s tradition or conventional medicine and are not fully integrated into the dominant healthcare system. They are used interchangeably with traditional medicine in some countries [6]. So-called CAM was first defined as a heterogeneous group of health care practices such as Ayurvedic medicine and other Indian systems of medicine such as Yoga, varieties of Chinese medicine, homoeopathy, Swedish massage and/or Qi Gong [7]. In recent years, the National Center for Complementary and Integrative Medicine (NCCIH), formerly known as the National Center for Complementary and Alternative Medicine (NCCAM) in the United States, defined CAM as a group of diverse medical and health care systems, practices, and products that are not presently considered to be part of conventional medicine [8]. In Europe, the CAMbrella Project [9,10] defined CAM as a variety of different medical systems and therapies based on the knowledge, skills and practices derived from theories, philosophies and experiences used to maintain and improve health, as well as to prevent, diagnose, relieve or treat physical and mental illnesses. Despite their beginnings, in which CAM therapies were mainly used outside conventional health care, many countries have now or are adopting and adapting some NPIs by conventional health care [9,10].

## 2. An Example of Existing Research on NPIs for Pain Management

During the last decade, multiple studies have demonstrated the effectiveness of NPIs in improving chronic pain management and outcomes, including, for example, physical therapy [11,12], cognitive behavioural therapy [13,14,15], mindfulness-based stress reduction [16], yoga [17,18], and chiropractic treatment [19]. While medication-only treatment strategies may foster passive coping styles, NPIs benefits may be achieved in part through a reinforcing cycle of patient empowerment and self-efficacy, fostering active problem-solving, a more realistic goal setting and a functional/rehabilitative outlook [20,21]. Despite all these benefits in this field, as happens with other health conditions and settings, several barriers to its implementation still exist.

Awareness and knowledge-related factors, as well as treatment belief-related factors, revealed several fundamental issues that, considered together, suggested a need for designing and implementing broad-based, multi-pronged strategies. In this sense, it has been reported that a significant proportion of providers and patients are still sceptical about certain NPIs, and sometimes might not fully understand their rationale nor what array of evidence-based NPIs exist for each health condition. Academic detailing, in which providers are specifically trained about treatment strategies, could be one approach to enhance this education [22]. However, some research suggests that targeting provider education alone would be insufficient since patients’ attitudes and preferences have been also identified as barriers. This suggests that provider and staff training in the communication and education (of patients) about the multimodal pain treatment philosophy is needed. Furthermore, this lack of awareness of NPIs’ availability suggested the need for designing and studying the effects of advertising campaigns, leveraging the potentialities of mass media and new technologies, employing a broad-based promotion of the multimodal and multidisciplinary treatment paradigm, which may help support cultural change. Australia’s “*Back Pain: Don’t Take It Lying Down*” campaign was a successful example [23]. Several inaccurate but commonly held treatment beliefs, for example, that NPIs cannot or should not be used if patients are experiencing stress or other significant medical issues, could be specific targets for motivational enhancement and educational messages. Embedded in addressing treatment beliefs and increasing knowledge and awareness of NPIs is the need to improve patient-provider interactions. Distrust in providers and patients’ lack of motivation to engage in NPIs suggest that training providers in more effective communication techniques are important. Motivational interviewing strategies [24] and other pain communication strategies such as validation [25] are needed to help providers more effectively engage with patients with chronic pain. Similarly, lack of support from medical providers, peers, friends, and family was identified as a potential barrier to NPIs utilisation, suggesting that support is needed for successful engagement in NPIs. Indeed, encouragement from the medical team has been repeatedly identified as one of the most important facilitators of NPIs’ engagement [26].

## 3. Final Remarks

As stated before, the definition of non-pharmacological is any intervention intended to improve the health or the well-being of individuals that do not involve the use of any drugs or medicine. Healthcare providers and users might lack education or awareness about the benefits of NPIs for different health conditions. This situation could be explained because there is still a lack of rigorous and well-designed research studies providing evidence-based results about NPIs’ effectiveness and efficacy, but also because communication and dissemination of such benefits are still insufficient or inadequate. Including multiple stakeholder groups led to a robust array of factors that could serve as targets for designing and developing interventions to improve the design, test and uptake of these non-pharmacological evidence-based interventions [27].
